# Function of nuclear transport factor 2 and Ran in the 20E signal transduction pathway in the cotton bollworm, *Helicoverpa armigera*

**DOI:** 10.1186/1471-2121-11-1

**Published:** 2010-01-02

**Authors:** Hong-Juan He, Qian Wang, Wei-Wei Zheng, Jin-Xing Wang, Qi-Sheng Song, Xiao-Fan Zhao

**Affiliations:** 1 School of Life Sciences, the Key Laboratory of Plant Cell Engineering and Germplasm Innovation, Ministry of Education, Shandong University, Jinan 250100, Shandong, PR China; 2 Division of Plant Sciences, University of Missouri, Columbia, Missouri 65211, USA

## Abstract

**Background:**

Nuclear transport factor 2 and small GTPase Ran participate in the nucleo-cytoplasm transport of macromolecules, but their function in the 20-hydroxyecdysone (20E) signal transduction pathway are not well known.

**Results:**

A 703 bp encoding Ntf2 and a 1233 bp encoding Ran full-length cDNAs were cloned from *Helicoverpa armigera*, and named *Ha-Ntf2 *and *Ha-Ran*, respectively. Northern blot and immunoblotting revealed that *Ha-Ntf2 *had an obviously higher expression levels in the head-thorax and integument of the metamorphically committed larvae. In contrast, the expression of Ha-Ran did not show obvious variation at various developmental stages in four tissues by immunoblotting analysis, except in the midgut, which showed increased expression from 5th-36 h (molting) to 6th-48 h. Both expressions of *Ha-Ntf2 *and *Ha-Ran *could be upregulated by 20E *in vitro*. Immunohistochemistry revealed that Ha-Ntf2 and Ha-Ran were primarily localized in the nucleus of various tissues. Protein binding assay and co-immunoprecipitation indicated that Ha-Ntf2 and Ha-Ran can combine with each other *in vitro *and *in vivo*. Knock down of *Ha-Ntf2 *or *Ha-Ran *by RNAi resulted in the suppression of other 20E regulated genes including *EcR-B1*, *USP1*, *E75B*, *BR-CZ2*, *HHR3 *and *Ha-eIF5c*. In addition, the knockdown of *Ha-Ntf2 *resulted in Ha-Ran being prevented in the cytoplasm. The nuclear location of the ecdysone receptor b1 (EcR-B1) was also blocked after the knockdown of *Ha-Ntf2 *and *Ha-Ran*.

**Conclusion:**

These evidences suggested that Ha-Ntf2 and Ha-Ran participated in the 20E signal transduction pathway by regulating the location of EcR-B1.

## Background

Molecules are transported into or out of the nucleus in two different ways, passive diffusion and active transport. Smaller molecules (<40 kDa) diffuse passively through the nuclear pore complex (NPC). Macromolecules, such as cargo proteins, require some soluble nucleic or cytosolic factors for active transport [1,2]. This nucleocytoplasmic transport not only locate proteins in the cytoplasm or nucleus through export ribosomes, mRNAs and tRNAs to the cytoplasm, or import nuclear proteins from the cytoplasm[3], but also functions as a key step in signal transduction pathways and in the regulation of cell cycle progression [4]. The export of mRNA is not dependent of members of the conventional nuclear export family, exportins and the Ran GTPase [5,6]. Nuclear transport factor 2 (Ntf2) and Ran were originally identified as soluble cytosolic factors necessary for the efficient protein transport in permeabilized mammalian cells [7]. Ntf2 interacts with the cytosolic factor Ran to perform a function in the nuclear transport [1]. In mammalian cells, Ntf2 was initially identified as stimulating the import of proteins into the nucleus [8], particularly resulting in the accumulation of Ran in the nucleus [9].

Ran belongs to the small Ras GTPase superfamily and switches between the GDP-bound (RanGDP) and GTP-bound (RanGTP) states for its activity [10,11]. RanGDP combines with Ntf2 and is transported into the nucleus. The Ran nucleotide exchange factor (RCC1) converts RanGDP into RanGTP in the nucleus [11,12]. RanGTP is required to release the imported cargo proteins from the importin proteins (importinα and importinβ) by competitive binding to importin β in the nucleus [10]. RanGTP is then transported from the nucleus into the cytoplasm by importinβ and is converted to RanGDP by RanGAP, a cytoplasmic GTPase-activating protein [13]. This cause Ran to act as a major regulator of nucleocytoplasmic transport and to regulate the interaction between proteins.

The Ntf2 protein binds specifically to the Ran-GDP form [14] and then is transported into the nucleus [15]. A mutant Ntf2 that cannot bind Ran is unable to facilitate Ran import into the nucleus [16]. Ntf2 is an essential protein in yeast and *Caenorhabditis elegans *and nuclear protein import was destroyed by its effective negative mutants. For example, the conditional alleles of yeast *ntf-2 *show defects in nuclear protein import [17,18]. Also, loss of Ntf2 using antibodies prevents the nuclear import of proteins in HeLa cells [15]. In *Drosophila*, mutants of Ntf2 affect the import of Rel proteins to nuclear in the immune response and show a specific eye phenotype [19]. The overexpression of Ntf2 disturbs the nuclear import in a Ran-binding-dependent manner in *Arabidopsis *[20]. Interaction between Ntf2 and Ran is necessary for the nuclear import of the filamentous actin capping protein, CapG [21].

In holometabolous insects, the life cycle is characterized by a series of moltings, including larval molting (molting) and metamorphic molting (metamorphosis), which are regulated by ecdysteroids (20-hydroxyecdysone, 20E) and the juvenile hormone (JH). Ecdysteroids orchestrate the molting process and JH determines the nature of the molt [22]. JH is normally present during the larval stages to enable growth and progression from one larval stage to the next until the larva reaches the appropriate size for metamorphosis [22]. Metamorphosis is regulated by changes in the titer of the steroid hormone 20E when the amount of JH decreases. A pulse of 20E at the end of the last larval stage triggers the onset of prepupal development [23]. 20E regulates the expression of some early response genes, such as the transcripition factors *Broad-Complex *(*BR-C*), *E74 *and *E75 *[24,25] through the 20E receptors, the heterodimer nuclear hormone receptors of the ecdysone receptor (EcR) and the ultraspiracle (Usp) [26,27]. These transcription factors then induce the expression of other late genes [28]. During the 20E signal transduction, proteins are frequently imported into or exported from nucleus. To date, however, little is known about the roles of Ntf2 and Ran in the proteins transporting involved in the 20E signal transduction pathway during insect molting or metamorphosis.

In previous work, we used the suppression subtractive hybridization (SSH) technique and detected an increased expression of *Ntf2 *during metamorphosis in *H.armigera*[29]. To demonstrate the role of Ntf2 in the 20E signal transduction pathway, we therefore cloned this gene and named it *Ha-Ntf2*. Since Ran is a cofactor of Ntf2, we also cloned Ran from *H. armigera *and named it *Ha-Ran*. We further investigated the expression patterns of *Ha-Ntf2 *and *Ha-Ran *in developmental stages and demonstrated the interaction of Ha-Ntf2 and Ha-Ran *in vitro *and *in vivo*. Meanwhile, we studied the 20E regulation of these two genes. Furthermore, using RNAi technique in the *H. armigera *epidermis (HaEpi) cell line, we found that after interfering *Ha-Ntf2 *and *Ha-Ran*, the expression of other genes, such as ecdysone receptor b1 (*EcR-B1*), ultraspiracle protein 1 (*USP1*), ecdysone induced protein E75b (*E75b*), broad-complex Z2 (*BR-CZ2*), and hormone receptor 3 (*HHR3*) decreased. The distribution of Ha-Ran was prevented in the cytoplasm when *Ha-Ntf2 *was knocked down in the HaEpi cell, and the nuclear location of the EcR-B1 was also blocked after the knockdown of *Ha-Ntf2 *or *Ha-Ran*. These evidences suggested that Ha-Ntf2 and Ha-Ran participated in the 20E signal transduction pathway by regulating the location of EcR-B1.

## Results

### cDNA cloning and sequence analysis of Ha-Ntf2 and Ha-Ran

The full-length of *Ha-Ntf2 *and *Ha-Ran *cDNA were cloned using the strategies described in Materials and Methods section. The full length of *Ha-Ntf2 *was 703 bp, including a 16 bp 5' untranslated region (UTR), a 393 bp ORF and a 291 bp 3' UTR. The ORF encoded a 131- amino acid protein with a molecular weight of 14.7 kDa and a predicted theoretical isoelectric point (pI) of 4.7. The predicted protein had no signal peptide and 10-124 amino acids (aa) were predicted to comprise a Ntf2 domain. It included two predicted casein kinase II phosphorylation sites (aa 20-23 and 72-75) [see Additional file 1].

The complete cDNA of *Ha-Ran *included a 642 bp ORF, a 58 bp upstream sequence of ORF and a 533 bp downstream sequence. The cDNA encoded a 24.3 kDa protein with a pI of 6.96. 62-77 aa were predicted to be the GTP-binding nuclear protein Ran signature. The protein included six presumed protein kinase C phosphorylation sites, such as aa 7-9, 18-20, 51-53, 90-92, 94-96 and 147-149. 14-21 aa were predicted to constitute one ATP/GTP-binding site motif A (P-loop) [see Additional file 2].

Database searches using the BLASTX program revealed that *Ha-Ntf2 *has 80, 75, 68 and 68% identity to *Apis mellifera Ntf2*, *Aedes aegypti Ntf2*, *Maconellicoccus hirsutus Ntf2*, and *Drosophila melanogaster Ntf2*, respectively [see Additional file 3, A]. The sequence identity of *Ha-Ran *with other known insects such as *Bombyx mori*, *A. mellifera*, *A. aegypti *and *D. melanogaster *is 98, 95, 93 and 91%, respectively [see Additional file 3, B]. The alignment of *Ha-Ntf2 *and *Ha-Ran *sequences from other insects indicated that they are both highly conserved among different insects [see Additional file 3].

### Recombinant expression of Ha-Ntf2 and Ha-Ran

The Ha-Ntf2 and Ha-Ran proteins were expressed as soluble protein in *E. coli *BL21 (DE3) cells with pET30a (+)-Ha-Ntf2 or pET30a (+)-Ha-Ran as the expression vector. The target recombinant proteins His-rHa-Ntf2 and His-rHa-Ran were then purified to homogeneity using a Ni2^+^-NTA affinity column. The apparent molecular mass of the purified His-rHa-Ntf2 protein was about 21 kDa, consistent with what we expected. The predicted molecular mass of the Ha-Ntf2 protein was 14.7 kDa, plus about 6 kDa from the additional amino acids in the N-terminal of the expressed fusion protein on the vector [see Additional file 4, A]. The apparent molecular mass of the purified His-rHa-Ran protein was 35 kDa, this was higher than the theoretical weight of 30 kDa (24 kDa Ha-Ran, plus 6 kDa protein on the vector) [see Additional file 4, C]. This was because of the tag from the vector; when we hydrolyzed the purified His-rHa-Ran protein using thrombin, the molecular mass of rHa-Ran was 24 kDa (see later the result described in the binding assay). The polyclonal antibodies against Ha-Ntf2 or Ha-Ran were tested by immunoblotting using the proteins from 5-36 h fat bodies and results showed the polyclonal antibodies were specific [see Additional file 3, B, D].

### Expression profiles of Ha-Ntf2 and Ha-Ran during developmental stages

**Figure 1:**
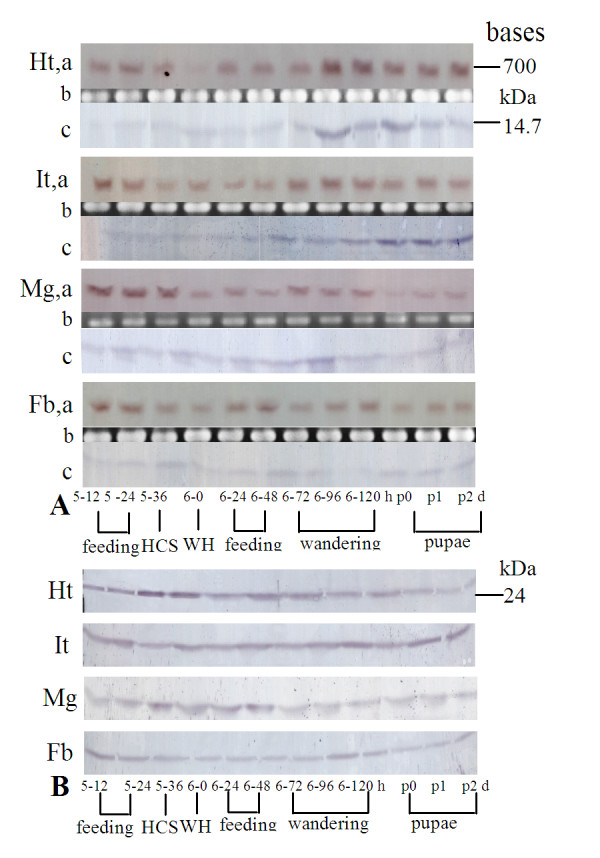
**Expression profiles of Ha-Ntf2 and Ha-Ran in four tissues samples during developmental stages**. A, Ha-Ntf2: a, Northern blot; b, 18s rRNA; c, immunoblotting. B, Ha-Ran: analyzed by immunoblotting. 5-12, 5-24, 5-36 (HCS), 6-0 (WH),6-24,6-48,6-72 (wandering d 1), 6-96 (wandering d 2) and 6-120 h (prepupae or quiescent d 1) indicated the different times of larvae; p0, pre-pupae; p1, 1st d pupae; p2 d, 2nd d pupae; Ht: head-thorax; It: integument; Mg: midgut; Fb: fat body; HCS, head capsule slippage; WH, white head capsule.

The expression profiles of *Ha-Ntf2 *throughout developmental stages were further analyzed in the head-thorax, integument, midgut and fat body from 5th-12 h to pupae 2 d using northern blot analysis. Results showed that the expression of *Ha-Ntf2 *transcription in the head-thorax was consistent from 5th-12 h feeding larvae to 6th-72 h (wandering d 1) larvae except 6th-0 h (WH, white head, newly ecdysed), but it was obviously upregulated from 6th-96 h (wandering d 2) larvae to p2 d (2 day after pupation) pupae. The expression of *Ha-Ntf2 *in the integument was similar to that in the head-thorax. However, the transcription of *Ha-Ntf2 *in the midgut was not regular; it was higher from 5th-12 h feeding stage to head capsule slippage (HCS) molting stage. It then decreased after the larvae entered the 6th instar, and it was upregulated slightly in the wandering stage (6th-72 h to 6th-120 h), and then downregulated when the larvae entered the pupal stage, although the protein translation seemed regular, it was upregulated in the wandering stage. In the fat bodies, *Ha-Ntf2 *transcription did not differ much during various developmental stages (Figure 1A). In contrast to the expression of *Ha-Ntf2*, immunoblotting analyses indicated the expression profiles of Ha-Ran in the four tissues had relatively even expression levels at various developmental stages compared with *Ha-Ntf2*, except for some increases in head-thorax during larval molting (5th-36 h to 6th-48 h) (Figure 1B).

### Location of Ha-Ntf2 and Ha-Ran in the tissues

**Figure 2:**
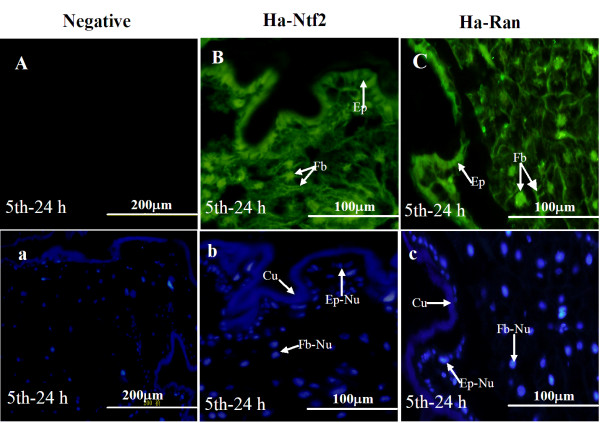
**Immunohistochemical location of Ha-Ntf2 and Ha-Ran in the integument and fat body of the feeding 5th instar larva (5th-24 h)**. Panel A, negative controls with preserum replacing antiserum; panel B, same specimens stained by anti-Ha-Ntf2; panel C, stained by anti-Ha-Ran; panels a, b, c are the same as A, B and C, respectively, but stained with DAPI. Fb, fat body; Ep, epidermis; Cu, cuticle; Fb-Nu or Ep-Nu: the nucleus of the fatbody or epidermis, respectively.

We took integument and fat body samples from 5th-24 h larvae to localize Ha-Ntf2 or Ha-Ran in the tissues. The Ha-Ntf2 signal was detected in the epidermis and fat body; the nuclei in the fat body released an intensive signal (Figure 2B). The results showed that Ha-Ran was also predominantly located in the nucleus, especially in the fat body (Figure 2C).

### Interaction of Ha-Ntf2 and Ha-Ran in vitro or in vivo

**Figure 3:**
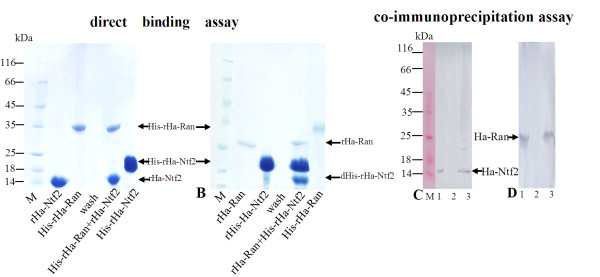
**Binding assay and co-immunoprecipitation assay to show the interaction between Ha-Ntf2 and Ha-Ran *in vitro *or in *vivo***. **A, B**: SDS-PAGE to show the direct binding assay between the rHa-Ntf2 and rHa-Ran *in vitro*. **A**, rHa-Ntf2 binds on His-rHa-Ran affiliated on Ni2^+^-NTA column. **B**, rHa-Ran binds on His-rHa-Ntf2 affiliated on Ni2^+^-NTA column. rHa-Ntf2 or rHa-Ran: removed His-tag by thrombin; dHis-rHa-ntf2: the degradation of His-rHa-ntf2 in the process of binding. **C, D**: immunoblotting to examine the interaction of Ha-Ntf2 and Ha-Ran produced by co-immunoprecipitation. **C**, Ha-Ntf2 detection from the co-immunoprecipitation (co-ippt) produced by anti-Ha-Ran antibody. Lane 1, protein extracts from the whole larvae of 6th-96 h; lane 2, sample from the last wash of the co-ippt; lane 3, eluted proteins from well-washed co-ippt produced by anti-Ha-Ran antibody. **D**, Ha-Ran detection from the co-ippt produced by anti-Ha-Ntf2 antibody. Lanes 1 and 2 are the same as in C; lane 3, eluted proteins from well-washed co-ippt produced by anti-Ha-Ntf2 antibody. M, protein marker.

To examine the interaction of Ha-Ntf2 and Ha-Ran *in vitro*, we performed direct binding assay *in vitro*. In the binding assay, after the His-rHa-Ran affiliated Ni2^+^-NTA column that was bonded by rHa-Ntf2 was washed to clear, both rHa-Ntf2 and His-rHa-Ran were detected in the eluted proteins from the column (Figure 3A). Similarly, both rHa-Ran and His-rHa-Ntf2 were detected from the His-rHa-Ntf2 affiliated Ni2^+^-NTA column that was bounded by rHa-Ran (Figure 3B), although His-rHa-ntf2 was partially degraded. These results suggested that rHa-Ran and rHa-Ntf2 could bind with each other *in vitro*.

To examine the interaction of Ha-Ntf2 and Ha-Ran *in vivo*, we performed a co-immunoprecipitation assay. Results showed that Ha-Ntf2 was detected in the precipitate produced by anti-Ha-Ran (Figure 3C, lane 3). Similarly, Ha-Ran was detected in the precipitate produced by anti-Ha-Ntf2 (Figure 3D, lane 3). These experiments suggested that Ha-Ntf2 and Ha-Ran bond together *in vivo*.

### Regulation of the Ha-Ntf2 or Ha-Ran by 20E

**Figure 4:**
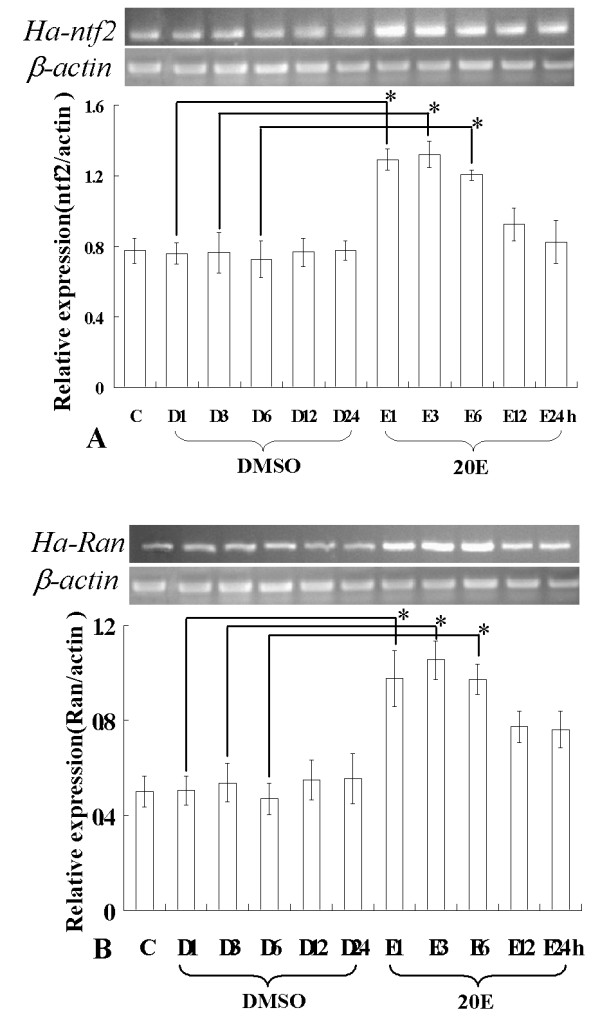
**Regulation of 20E in the expression of *Ha-Ntf2 *or *Ha-Ran***. A: RT-PCR analysis the regulation of 20E in the expression of *Ha-Ntf2*. B: Regulation of 20E in the expression of *Ha-Ran*. Other signs are: C stands for the normal 6th-0 h larvae; D1, D3, D6, D12 and D24 are 1, 3, 6, 12 and 24 h after injected by the DMSO, respectively; E1, E3, E6, E12 and E24 stand for 1, 3, 6, 12 and 24 h after injected by the 20E, respectively. *β-actin *is used as a quantitative control. Error bars represent the standard deviation in three independent experiments. An asterisk indicates significant differences (Student's t-test, *: p < 0.05).

The cDNA synthesized by total RNA, which was isolated from the larvae after injection of 20E, was used for analysis. Semi-quantitative RT-PCR results suggested that *Ha-Ntf2 *or *Ha-Ran *were distinctly upregulated from 1 to 3 h after the injection of 20E. This is compared to the expression in the normal 6th-0 h larvae and the larvae injected with DMSO. The expression of *Ha-Ntf2 *and *Ha-Ran *started to decline from the 12 h (Figure 4).

### Function of Ha-Ntf2 and Ha-Ran in the 20E signal pathway

**Figure 5:**
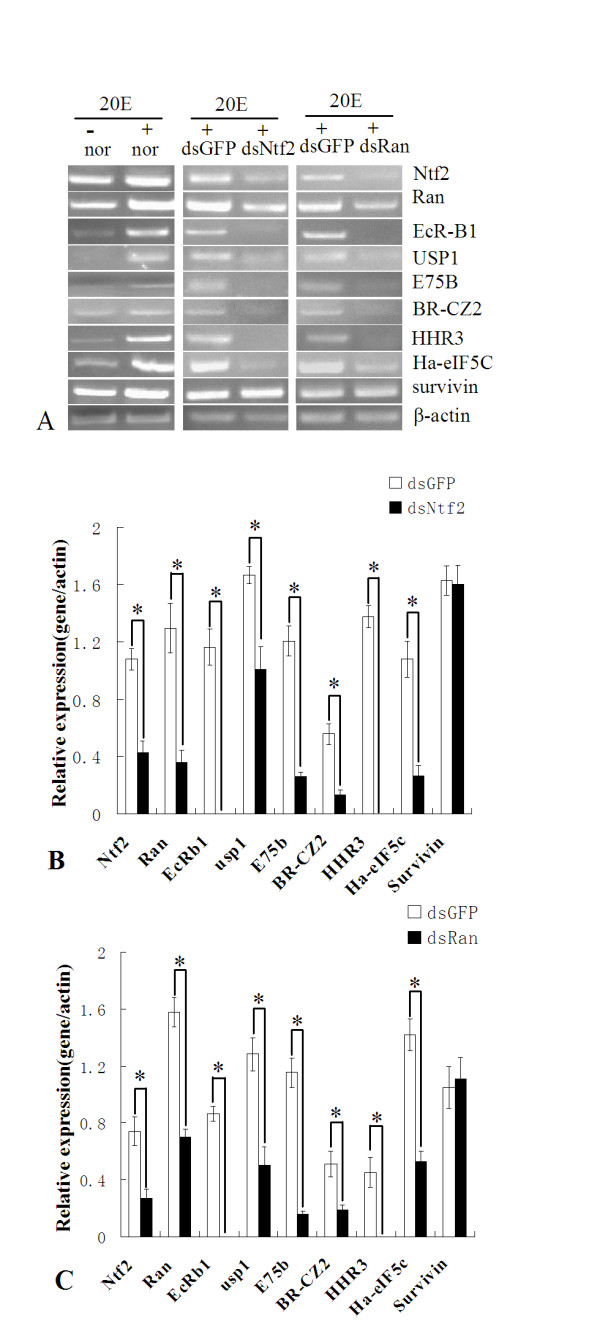
**Effects on the genes in the HaEpi cell after the knockdown of *Ha-Ntf2 *or *Ha-Ran***. A. RT-PCR analysis the expressions of other genes in the normal cell, the cells plus 20E, the cells with dsGFP added, dsNtf2 and dsRan, respectively. B: Statistical analysis of results in after the knockdown of *Ha-Ntf2 *comparing to the control adding with the dsGFP. C: Statistical analysis of results in after the knockdown of *Ha-Ran *comparing to the control adding with the dsGFP. *β-actin *is used as a quantitative control. Error bars represent the standard deviation in three independent RNAi experiments. Asterisks stand for statistically significant differences. (*: p < 0.05 by T-test, n = 3), respectively.

To examine the function of Ha-Ntf2 and Ha-Ran in the 20E signal pathway, we examined the mRNA levels of genes involved in the 20E signal pathway using RT-PCR. Compared to the control cells without 20E, the expression of genes, including *Ha-Ntf2*, *Ha-Ran*, *EcR-B1*, *USP1*, *E75B*, *BR-CZ2*, *HHR3*, *Ha-eIF5c *and *survivin*, were all upregulated after induction by 20E. The expression of *Ha-Ntf2 *was obviously knocked down after adding *dsNtf2*. The expression of other genes, including *Ha-Ran*, *EcR-B1*, *USP1*, *E75B*, *BR-CZ2*, *HHR3 *and *Ha-eIF5c*, also decreased when compared to their mRNA levels in the cells with dsGFP (Figure 5A, B). Similarly, the knockdown of *Ha-Ran *also resulted in its downregulation and the downregulation of other genes, including *Ha-Ntf2*, *EcR-B1*, *USP1*, *E75B*, *BR-CZ2*, *HHR3 *and *Ha-eIF5c*. The expression of *survivin *was not affected after the knockdown of *Ha-Ntf2 *or *Ha-Ran *(Figure 5A, C).

**Figure 6:**
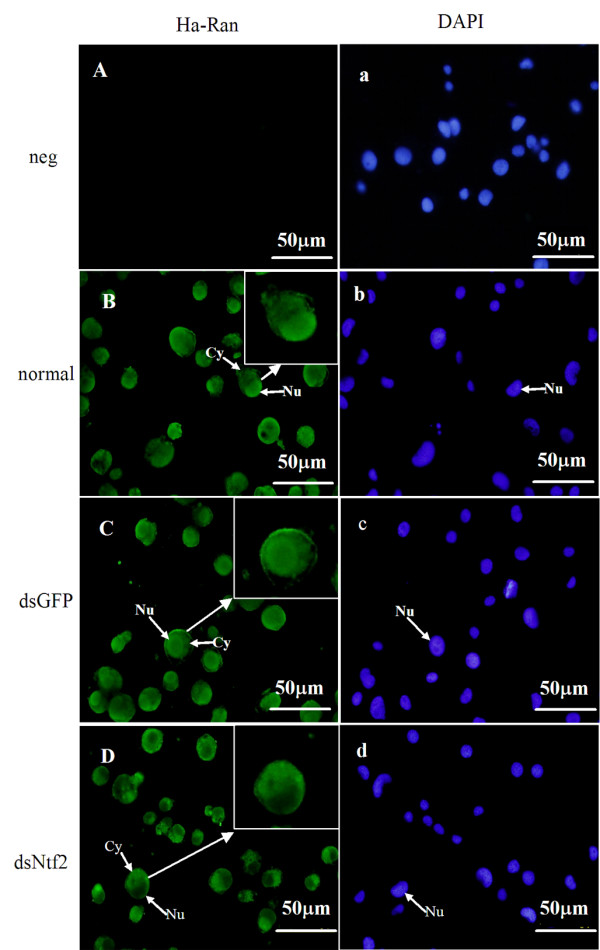
**The location of Ha-Ran in the HaEpi cells after knock down of *Ha-Ntf2***. The cells were immunostained with polyclonal antibody against Ha-Ran (green color). Nuclei were counterstained with DAPI (blue color). Arrow indicates the magnified cell in B, C and D, respectively. Nu: stand for the nucleus; Cy:cytoplasm. Size bars = 50 μm.

To demonstrate the mechanism of the above results, we further examined the variation of subcellular localization of *Ha-Ran*, *EcR-B1 *and *USP1*. Results showed that Ha-Ran protein was mainly located in the nucleus of normal cells (Figure 6B) or the cells with dsGFP added (Figure 6C), whereas its signal was increased in the cytoplasm after the knockdown of *Ha-Ntf2 *(Figure 6D).

**Figure 7:**
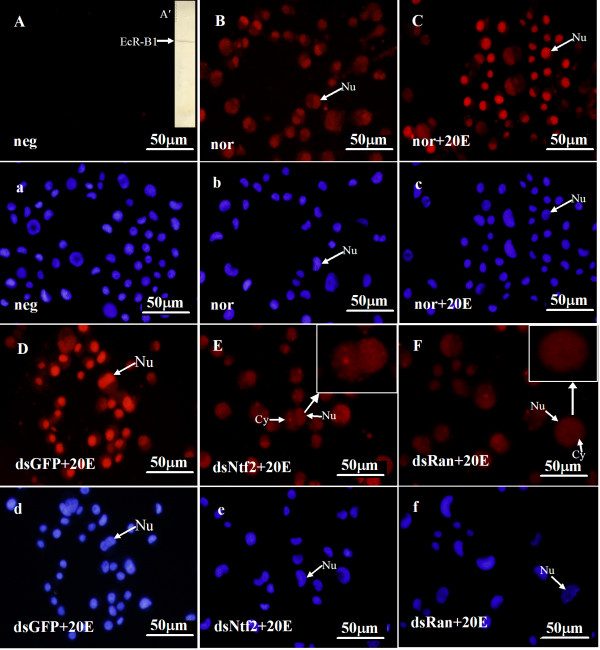
**Variation of the subcellular localization of EcR-B1 in the HaEpi cell after the knockdown of *Ha-Ntf2 *or *Ha-Ran***. Panel A': The EcR-B1 from the HaEpi cell extract could be detected by the monoclonal antibody of EcR-B1 from the *M. sexta *using immunoblotting. Panels A-F: The red color indicates EcR-B1 stained with anti-EcR-B1 monoclonal antibody; the blue color indicates nuclei counterstained with DAPI. Neg, negative control without first antibody; nor, normal cells cultured in Grace's medium; nor+20E, the cells cultured in Grace's medium plus 20E; dsGFP+20E, the cells cultured in Grace's medium plus dsGFP and 20E; dsRan+20E, the cells cultured in Grace's medium plus dsRan and 20E; dsNtf2+20E, the cells cultured in Grace's medium plus dsNtf2 and 20E. Nu: nucleus; Cy: cytoplasm. Size bars = 50 μm.

**Figure 8:**
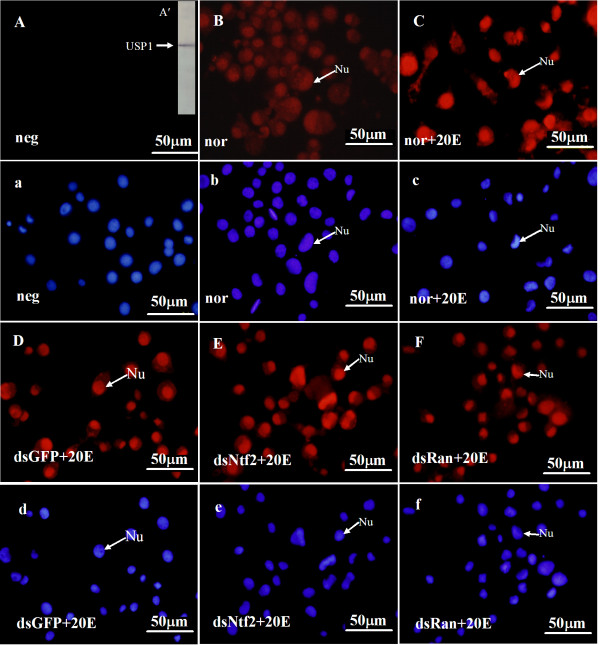
**The subcellular localization of USP1 in the HaEpi cell after the knockdown of *Ha-Ntf2 *or *Ha-Ran***. Panel A': The USP1 from the HaEpi cell extract could be detected by the monoclonal antibody of USP1 from the *D*. *melanogaster *using the immunoblotting. The red color indicates USP1 stained with anti-USP1 monoclonal antibody; the blue color indicates nuclei counterstained with DAPI. Nu: nucleus; Cy: cytoplasm. Size bars = 50 μm.

Similar to the variation of Ha-Ran in the cytoplasm and nucleus, EcR-B1 was mainly located in the nuclei of normal cells, normal cells with 20E or cells with dsGFP and 20E (Figure 7B, C, D). However, the EcR-B1 signal was increased in the cytoplasm after the knockdown of *Ha-Ntf2 *or *Ha-Ran*, but not after injection of dsGFP (Figure 7D-F). However, the distribution of USP1 was primarily located in the nucleus and there were no obvious changes after the knockdown of *Ha-Ntf2 *or *Ha-Ran *(Figure 8D-F).

## Discussion

In this study, we cloned the cDNA of *Ha-Ntf2 *and *Ha-Ran *from the cotton bollworm, *H. armigera*. Homology analysis revealed that *Ha-Ntf2 *and *Ha-Ran *were conserved among different insect species. The expression profiles in different tissues at various developmental stages and ecdysone hormone injection experiments suggested that *Ha-Ntf2 *was regulated by 20E. However, *Ha-Ran *was likely a housekeeping gene but could be regulated by 20E *in vitro*. Ha-Ntf2 and Ha-Ran could combine together *in vitro *and *in vivo*. The nuclear location of Ha-Ran was prevented after the knockdown of *Ha-Ntf2*, which in turn blocked the nuclear transport of EcR-B1 in the cells. Both of *Ha-Ntf2 *and *Ha-Ran *are needed in the 20E signal transduction pathway through participating in the nuclear transport of EcR-B1 in the cells.

Our studies have shown that Ha-Ntf2 is expressed in the head-thorax, integument, midgut and fat body, but their expression profiles in these tissues at the various developmental stages were different. The expressions of *Ha-Ntf2 *in the head-thorax and the integument were obviously upregulated in the metamorphically committed larvae. The head-thorax contain major integument, in addition, they also contain the brain, prothoracic gland, foregut, fat body, and other organs. Since the expression pattern was similar to the integument, the mRNA detected in the head-thorax might major from integument in this part. From the 6th-48 h, the larvae stopped eating and entered the wandering stage. Then, the larvae morphologically and physiologically transformed into pupa (metamorphosis). The up-regulation of Ha-Ntf2 that occurred during the prepupal period was likely due to the increased ecdysteroid necessary for pupation. Similarly, in the midgut, the increased expression of *Ha-Ntf2 *in the 5th instar larvae and metamorphic larvae might have the function in protein transport.

Different from Ha-Ntf2, Ha-Ran did not show obvious variation in the different tissues during larval development, this suggests that Ha-Ran is likely a housekeeping gene and its function in transporting proteins into the nucleus might be regulated by its conversion between RanGTP and RanGDP. The constitutive expression pattern suggests that Ran not only plays a key role in nuclear import, but also has been implicated in a wide variety of intranuclear events, such as centrosome duplication, microtubule dynamics, chromosome alignment, kinetochore attachment of microtubules, RNA transcription and processing, and RNA export. This would explain its abundance and why it is accumulated inside the nucleus [8,10]. The results from tissues and the HaEpi cell line also demonstrated that Ha-Ran protein was located primarily inside the nucleus. Our results showed that after knocking down of *Ha-ntf2*, the Ha-Ran protein was prevented in the cytoplasm. This result is similar to that in the ntf2 mutant, the Ran protein couldn't be imported into the nucleus [9]. Immunohistochemical results showed that the Ha-Ntf2 was distributed predominantly in the nucleus. There was some distribution in the cytoplasm which was related to its migration between the cytoplasm and the nucleus. This phenomenon is different from the report that Ntf2 protein concentrated at the nuclear envelope [17,20].

*H. armigera *has similar development patterns to other lepidopteran insects [30]. The developmental expression patterns of *Ha-Ntf2 *are correlated to the 20E puff during molting and metamorphosis [31], suggesting that *Ha-Ntf2 *was upregulated by 20E *in vivo*. Our experiments *in vitro *indeed confirmed that *Ha-Ntf2 *was upregulated by 20E. This was also proved in the HaEpi cell line by Shao *et al *(2008).

The interaction between Ran and Ntf2 was previously proved using sepharose bead binding assay and pull down assays [14,18]. We also proved here that Ha-Ran and Ha-Ntf2 could bind with each other *in vitro *and *in vivo*. This evidence indicated that Ha-Ntf2 and Ha-Ran performed roles by interacting with each other. RNAi experiments suggested that interfering with any one of them resulted in a loss of the protein transport functions and the later events in signal transduction pathways. EcRb, USP1, E75B, Broad-complex and HHR3 are molting-related transcriptional factors and are upregulated by 20E [22,32]. *Ha-eIF5C*, *Ha-Ntf2 *and *Ha-Ran *are late genes in the 20E signal transduction pathway because they have been proved to be upregulated by 20E [33,34]. When the gene *Ha-Ntf2 *was suppressed, not only the expression of *Ha-Ran *decreased, but also the expressions of other genes involved in the 20E signal transduction pathway were affected, such as *EcR-B1*, *USP1*, *E75B*, *BR-C Z2*, *HHR3 *and *Ha-eIF5C*. Similarly, when the gene *Ha-Ran *was suppressed, the expression of *Ha-Ntf2*, *EcR-B1*, *USP1*, *E75B*, *BR-C Z2*, *HHR3 *and *Ha-eIF5C *were also affected. Only *survivin*, an apoptosis inhibitor gene, was not affected by the knockdown of both *Ha-Ran *and *Ha-Ntf2*. These results suggested that both *Ha-Ntf2 *and *Ha-Ran *are necessary for the transcription of these genes. The gene transcription needs the participation of transcription factors such as *EcR-B1*, *USP1 *and *E75B*, which were initiated by 20E in the signal transduction pathway, to transport into the nucleus. The evidence that the expression of 20E regulated genes decreased after the knockdown of *Ha-Ntf2 *or *Ha-Ran *suggested that *Ha-Ntf2 *and *Ha-Ran *are involved in the 20E signal transduction pathway.

**Figure 9:**
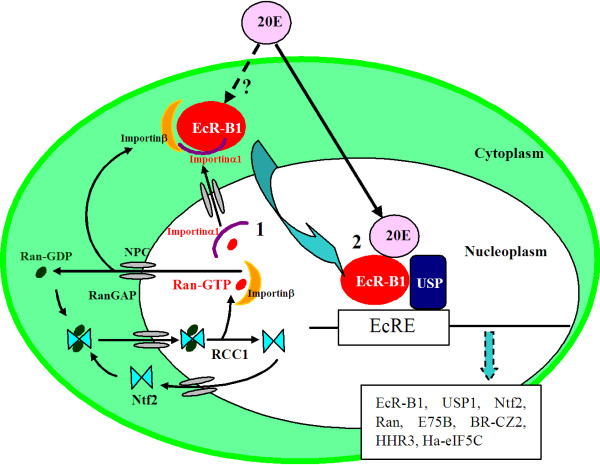
**Model of how the nuclear transport factor 2 and Ran may function in the import of the EcR-B1 in the 20E signal transduction pathway**. **1**, the EcR-B1 transport through the NPC by binding with the importinα1 and importinβ and goes into the nucleoplasm; **2**, in the nucleoplasm, 20E binds with EcR-B1 and formed trimeric complex with USP to regulate the transcriptions of genes. In the cytoplasm, NTF2 binds with RanGDP and transports RanGDP into the nucleoplasm, where RanGDP is converted into RanGTP by RCC1; RanGTP then is transported back into the cytoplasm by importinβ and hydrolyzed to RanGDP by RanGAP [44]; this process results in the energy gradient for the transport of EcR-B1 and the accumulation of EcR-B1 in the nucleus. **NPC**: nuclear pore complex; **RCC1**: Ran nucleotide exchange factor; **RanGAP**: Ran GTPase-activating protein. **EcRE**: ecdysone response elements.

The heterodimerization between EcR and USP is required for ligand and DNA binding and gene transcription [26,27,35]. EcR and USP can enter the nucleus separately and are mainly located in the nucleus [36,37]. The transport of EcR and USP of *D. melanogaster *from nucleus to cytoplasm was found in mammalian cells, which is a process that requires energy supplied by ATPase, not GTPase [38]. The gradient of RanGTP between nucleus and cytoplasm is critical for the directionality of nucleocytoplasmic transport of many cargos proteins by nuclear transport receptors [39]. The binding of RanGTP to importins not only dissociates nuclear import complexes, which lead to the nuclear accumulation of the cargo proteins containing nuclear localization signal (NLS), but also is required for the interaction of exportins with crago proteins which containing nuclear export signal (NES) [40]. The three known EcR isoforms (A, B1 and B2) all have two NESs in ligand binding domain and one NLS activity within the DNA-binding domain, and exhibit nucleocytoplasmic shuttling [41]. The location of EcR exhibits circadian rhythms, a daily rhythm in the nuclear abundance and subcelluar location in the epidermal cells of *Rhodnius prolixus*[42], which suggested that EcR plays its roles by travelling between the cytoplasm and the nucleus. EcR-B1 (cargo) is found to interact with importin α1 and exportin 1 [41]. Importin α1 and exportin 1 participate in the nucleocytoplasmic shuttling of proteins in mammalian cells [43]. Our results indicated that the nuclear location of Ha-Ran was prevented after the knockdown of *Ha-Ntf2*. In addition, the nuclear location of the 20E receptor EcR-B1 was also prevented after the knockdown of *Ha-Ntf2 *or *Ha-Ran*. Figure 9 showed that how the Ntf2 and Ran function in the import of the EcR-B1 in the 20E signal transduction pathway. The nucleocytoplasmic transport by impotinα and β referenced the schematic view reported by Isgro and Schulten [44]. After knock down of *Ha-Ntf2 *or *Ha-Ran*, the energy gradient of RanGTP between nucleus and cytoplasm was destroyed, therefore, the nuclear translocation of EcR-B1 was prevented, and thereby the 20E signal transduction pathway was blocked. However, the location of USP1 was not affected after the knockdown of *Ha-Ntf2 *or *Ha-Ran*.

## Conclusions

Ha-Ntf2 and Ha-Ran were primarily localized in the nucleus of various tissues and could interact with each other *in vitro *or *in vivo*. However, it was interesting to find that both of them could be upregulated by 20E. The nuclear location of Ha-Ran was prevented after the knockdown of *Ha-Ntf2*. The results that knocking down of *Ha-Ntf2 *or *Ha-Ran *resulting in the decrease of 20E regulated genes suggested that the transporting of the transcription factor EcR-B1 into the nucleus was prevented, so that the 20E signal transduction pathway was blocked. Therefore, Ha-Ntf2 and Ha-Ran participated in 20E signal transduction pathway by regulating the nuclear location of EcR-B1.

## Methods

### Insects

The cotton bollworm, *H. armigera*, was reared on an artificial diet in our laboratory in our laboratory and kept on a light-dark cycle of 14:10 h at 26°C [45].

### cDNA cloning of Ha-Ntf2 and Ha-Ran genes

A fragment of *Ha-Ntf2 *was obtained using suppression subtractive hybridization (SSH) [29], and a fragment of *Ha-Ran *was obtained from a random sequencing of the cDNA library. The full-length cDNA was cloned using 5'RACE and 3'RACE (Rapid-amplification of cDNA ends) techniques. The 5'end of the cDNA was amplified by a T3 primer (5'-aattaaccctcactaaaggg-3') and a reverse gene-specific primer Ntf2R (5'-tgcaactgtactccttcaaag-3'), RanR (5'-gcaggcatagcaacaaactccag-3') using the cDNA library as the template. The conditions were as follows: 1 cycle (94°C, 2 min); 35 cycles (94°C, 30 s; 51°C, 45 s; 72°C, 45 s); and one cycle (72°C, 10 min). Similarly, the 3' end of the gene was amplified using a gene-specific forward primer, Ntf2F (5'-cagcaatattacacactgttcg-3'), RanExpF (5'-tactcagaattcatggctgatgatatgcccaca-3'), and a 3' anchorR primer (5'-gaccacgcgtatcgatgtcgac-3') under the above mentioned PCR conditions. Functional sites were performed using ExPASy data bases http://www.expasy.org/prosite/.

### Recombinant expression and antibody preparation of Ha-Ntf2 and Ha-Ran

The *Ha-Ntf2 *cDNA (393 bp) and the *Ha-Ran *cDNA (642 bp) containing the *Eco*RI and *Xho*I sites were amplified, respectively. They were expressed in *E. coli *BL2 (DE3) using the pET30a (+) vector. Rabbit polyclonal antiserum against Ha-Ntf2 and Ha-Ran were prepared by affinity chromatography using purified recombinant protein. The specificity of the antiserum was examined by immunoblotting and the antiserum was used in the immunoassay experiments.

### Immunoblotting

Protein extracts (100 μg) of the *H. armigera *tissues were separated by SDS-PAGE, and transferred to a nitrocellulose membrane. Anti-Ha-Ntf2 or Ha-Ran was diluted 1:100 and the monoclonal anti-EcR-B1 or anti-USP1 was diluted 1:2000 in 2% non-fat milk in TBS (10 mM Tris-HCl, pH7.5; 150 mM NaCl), and the second antibody of horseradish peroxidase (HRP)-conjugated goat anti-rabbit IgG or HRP-was conjugated goat anti-mouse IgG diluted 1:10,000 in the same blocking buffer. The procedure has been reported previously [46].

### Northern blot analysis

Total RNA (10 μg) was extracted from various tissues during the developmental stages (from 5th-12 h to p2 d), and then was separated and transferred to a nylon membrane. The Dig-labeled RNA probes were prepared using a Dig-RNA labeling kit and Northern blot followed the method presented by Roche Company (Boehringer Mannheim, Germany).

### Immunohistochemistry

Integument and fat body from the feeding stage (5th-24 h) were excised and fixed in 4% paraformaldehyde in 1 × PBS (140 mM NaCl, 2.7 mM KCl, 10 mM sodium hydrogen phosphate, 1.8 mM potassium dihydrogen phosphate, pH 7.4) for 12 h at 4°C, then embedded in wax after dehydration. 4 μm cryosections were cut and placed on glass slides. The sections were then dried overnight. After rehydration, the slides were washed twice in 1 × PBS, and then incubated for 10 min in 0.2% Triton X-100/PBS, washed with PBS, and blocked with 2% bovine serum albumin (BSA) in PBS for 30 min at 37°C. Next, they were then incubated overnight at 4°C with anti-Ha-Ntf2 or anti-Ha-Ran (1:100 dilution in PBS) and then goat anti-rabbit-ALEXA 488 diluted to 1:1000 in 1 × PBS with 2% BSA at 37°C for 1 h. Nuclei were stained with DAPI for 10 min. Negative controls were treated in the same manner, but pre-immune rabbit serum was used in place of the antiserum against Ha-Ntf2 or Ha-Ran. Fluorescence was detected with an Olympus BX51 fluorescence microscope (Japan).

### Binding assay and Co-Immunoprecipitation

The bacterially expressed His-tag fusion proteins His-rHa-Ntf2 and His-rHa-Ran were purified using a nickel affinity column. Using thrombin, the His-tag was then cleaved from the fusion proteins at 22°C overnight and removed by dialyzing in 1 × PBS at 4°C overnight. Meanwhile, the His-rHa-Ntf2 and His-rHa-Ran were affiliated on the nickel affinity column. The column was washed repeatedly using 1 × PBS until no protein was detected. Then, His-tag free rHa-Ntf2 and rHa-Ran were added to the column to bind with His-rHa-Ran and His-rHa-Ntf2, respectively. After 2 h incubation, the column was washed again using 1 × PBS until the flow-through solution was washed out of the proteins. The proteins were then eluted off from the column using elution buffer and were detected using SDS-PAGE. The whole process was operated at 4°C.

In the co-immunoprecipitation assay, proteins were extracted from whole larvae of 6th-96 h using a binding buffer [10 μM Tris-HCl, pH 7.5; 2 μM β-mercaptoethanol; 1 μM EDTA (Ethylenediaminetetraacetic Acid), 10 mM PMSF (Phenylmethanesulfonyl fluoride)] and harvested by centrifugation at 4°C at 10,000 rpm for 20 min. Protein A Sepharose CL-4B beads (GE Healthcare) were added to the tube and equilibrated with binding buffer three times. Then, 0.3 ml prepared antibody and 0.3 ml 20 mM PBS (274 mM NaCl, 2.7 mM KCl, 20 mM sodium hydrogen phosphate, 3.5 mM potassium dihydrogen phosphate, pH 7.0, for the binding of IgG to the protein A beads) were then added, incubated with shaking at room temperature for 10 min, centrifuged to discard the supernatant, and washed with binding buffer 3 times for 2 min each time. 1 mg/ml protein were added and incubated at 4°C with shaking overnight. The precipitate was collected by centrifugation and washed with binding buffer three times. Proteins from Protein A Sepharose CL-4B beads were then eluted using citric acid buffer (0.1 M, pH 3.0) and were detected by immunoblotting using antibodies.

### Regulation of Ha-Ntf2 or Ha-Ran by 20E

The 6th instar 0 h (6th-0 h) larvae were injected with 20E (500 ng/5 μl/larvae), and the controls were injected same amount of DMSO (dimethyl sulphoxide) in PBS without the hormone. The total RNA was extracted together from 4 treated larvae by time intervals from 1 to 24 h using Unizol reagent. Five μg of the total RNA was used to reverse transcribe first-strand cDNA (First Strand cDNA Synthesis Kit, Sangon, Shanghai, China), for using as the PCR template. The PCR were amplified for different number of cycles, from 20 to 31, (sampled every three cycles) to make sure that the products of semi-quantitative RT-PCR were in the linear range of amplification of PCR. The Ha-ntf2 cDNA was amplified with the primers Ha-ntf2ExpF (5'-tactcagaattcatggcgctcaatccacaatac-3') and Ha-ntf2ExpR (5'-tactcactcgagattggcggctatgtcgtggat-3'); and the Ha-Ran cDNA was amplified with the primers Ha-Ran ExpF (5'-tactcagaattcatggctgatgatatgcccaca-3') and Ha-Ran ExpR (5'-tactcactcgagttacaagtcttcatcctcctc-3'). RT-PCR products were separated on 1% agarose gels and photographed under UV light with Quantity One software (Bio-Rad, Hercules, CA). The experiments were independently repeated three times, from injection of 20E to RT-PCR, and the data from 3 repeats of a template were statistically analyzed.

### RNAi in the HaEpi cell line and immunocytochemistry

The primers containing the T7 polymerase promoter sequence at the 5' end or 3' end were used to amplify the ORF of the *Ha-Ntf2 *or *Ha-Ran*. dsRNA of *Ha-Ntf2 *or *Ha-Ran *were then synthesized according to the Ambion MEGAscript RNAi kit. The HaEpi cell line was cultured according to the method established by our lab [33]. 5 μg dsRAN and 2.5 μl Lipofectamine 2000 mixture were diluted in 1 ml Grace's insect medium and directly added to the cells. After incubation at 26°C for 6 h, cells were rinsed and then re-fed with a normal medium containing 20E at 0.3 μM. After culturing for 6 h, total RNA were isolated from the cells for RT-PCR analysis. Cells adding with the equivalent amount dsGFP were prepared as the control.

Meanwhile, the HaEpi cells cultured as above were used for the immunocytochemistry. After adding dsRNA and incubation with 20E, the cells were fixed in freshly prepared 4% paraformaldehyde/PBS for 10 min and washed with 1× PBS (pH 7.4), then permeabilized in 0.2% Triton-X100/PBS for 10 min at room temperature. The cells were incubated with EcR-B1 or USP-specific monoclonal antibody diluted in blocking buffer in 1:1000 overnight at 4°C. The secondary antibody goat anti-mouse ALEX Fluor 568 was diluted in 1:1000 blocking buffer. The steps were the same as those described in the immunohischemistry.

## Accession numbers

The nucleotide sequence reported in this paper has been submitted to GenBank with accession number [GenBank: *Ha-ntf2*: DQ875254, *Ha-Ran*: EU860296.].

## Authors' contributions

Hong-Juan He performed the study. Qian Wang carried out the protein interaction. Wei-Wei Zheng preformed the culture of the HaEpi cell and the RNAi. Jin-Xing Wang and Qi-Sheng Song participated in the design and coordination of the work. Xiao-Fan Zhao conceived the study and helped to draft the final version of this manuscript. All authors read and approved the final manuscript.

## Supplementary Material

Additional file 1 **Full-length cDNA sequences and predicted amino acid sequences of *Ha-Ntf2***. Full-length cDNA sequences and predicted amino acid sequences of *Ha-Ntf2*. Amino acid residues in real line are casein kinase II phosphorylation sites. Click here for file

Additional file 2 **Full-length cDNA sequences and predicted amino acid sequences of *Ha-Ran***. Full-length cDNA sequences and predicted amino acid sequences of *Ha-Ran*. Overstriking amino acid residues are GTP-binding nuclear protein Ran signatures; Boxed amino acid residues are protein kinase C phosphorylation sites. The shadowed sequence is an ATP/GTP-binding site motif A. Poly A tail adding signal is in broad brush. Click here for file

Additional file 3 **Multiple alignments of *Ha-Ntf2 *and *Ha-Ran *with other insects**. Multiple alignments of *Ha-Ntf2 *and *Ha-Ran *with other insects. *D. melanogaster *[Ntf2, AAS98195.1; Ran: NP_651969.1]; *A. aegypti *[Ntf2, AAS79346.1; Ran, EAT38849.1]; *A. mellifera *[Ntf2, XP_392921.1; Ran, XP_393761.1]; *M. hirsutus *[ABM55654.1]; *B. mori *[NP_001040274.1]; *H. armigera *[Ntf2, DQ875254; Ran, EU860296]. The shadow regions in black are conserved sequences. Click here for file

Additional file 4 **Recombinant expression of Ha-Ntf2 and Ha-Ran in *E. coli *and specificity of antibodies**. Recombinant expression of Ha-Ntf2 and Ha-Ran in *E. coli *and specificity of antibodies. A and C, analysis of recombinant expression of Ha-Ntf2 and Ha-Ran by 12.5% SDS-PAGE; B and D, examining the specificity of the antibody against Ha-Ntf2 and Ha-Ran by immunoblotting, arrow indicate the target protein from 5-36 h fat bodies. Lane 1, *E. coli *proteins with pET30a-Ha-Ntf2 or pET30a-Ha-Ran without induction; lane 2, *E. coli *proteins with pET30a-Ha-Ntf2 or pET30a-Ha-Ran induced by IPTG; lane 3, lysate supernate; lane 4, lysate precipitate; lane 5, purified recombinant protein by nickel affinity chromatography; lane 6, standard protein marker. Click here for file
