# Supplementary figures and images for: Function of nuclear transport factor 2 and Ran in the 20E signal transduction pathway in the cotton bollworm, Helicoverpa armigera
Source: BMC Cell Biol. 2010 Jan 2;11:1. doi: 10.1186/1471-2121-11-1 (PMC2830935; doi:10.1186/1471-2121-11-1)

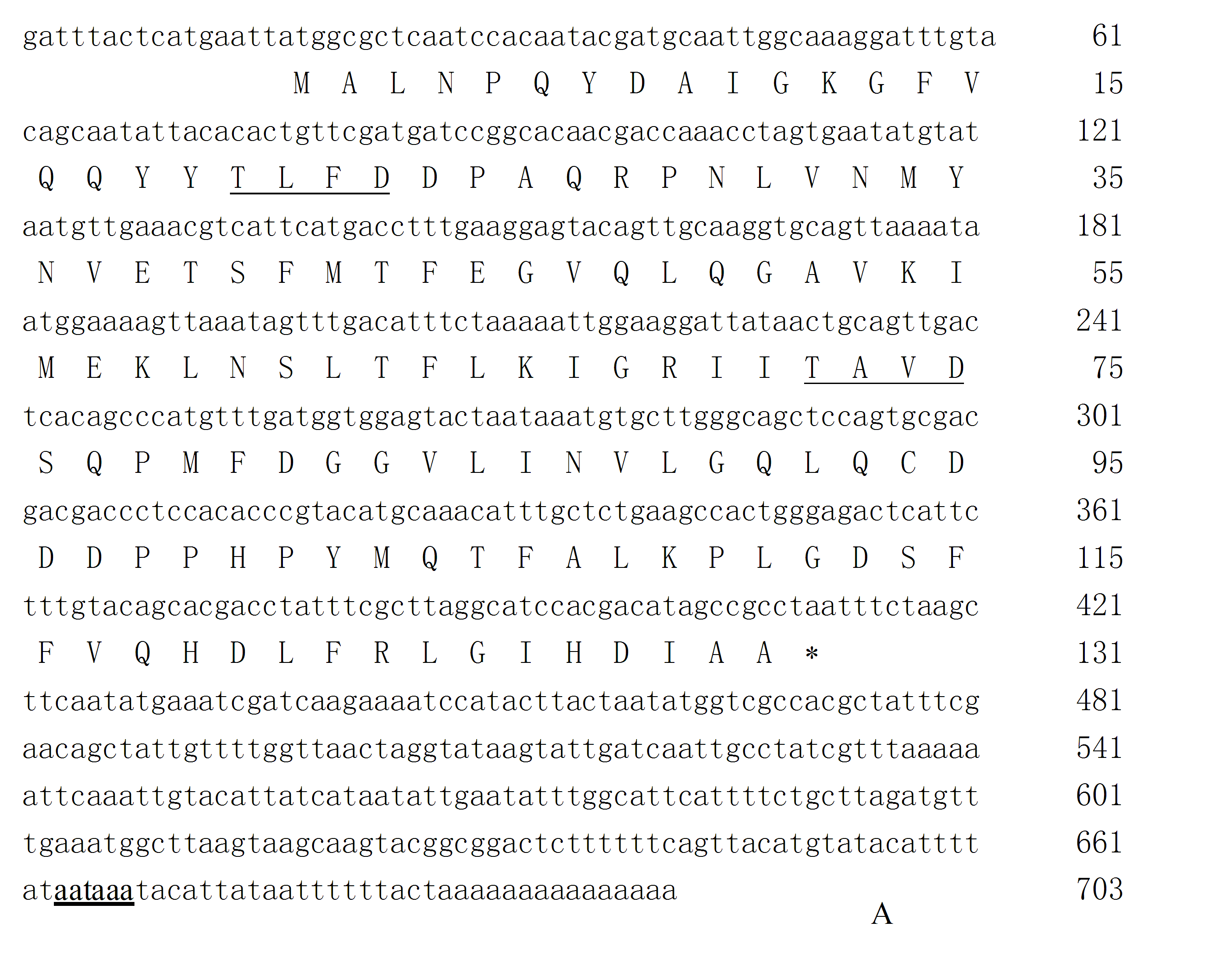

Supplement: Additional file 1 — Full-length cDNA sequences and predicted amino acid sequences of Ha-Ntf2. Full-length cDNA sequences and predicted amino acid sequences of Ha-Ntf2. Amino acid residues in real line are casein kinase II phosphorylation sites. [file 1471-2121-11-1-S1.TIFF]

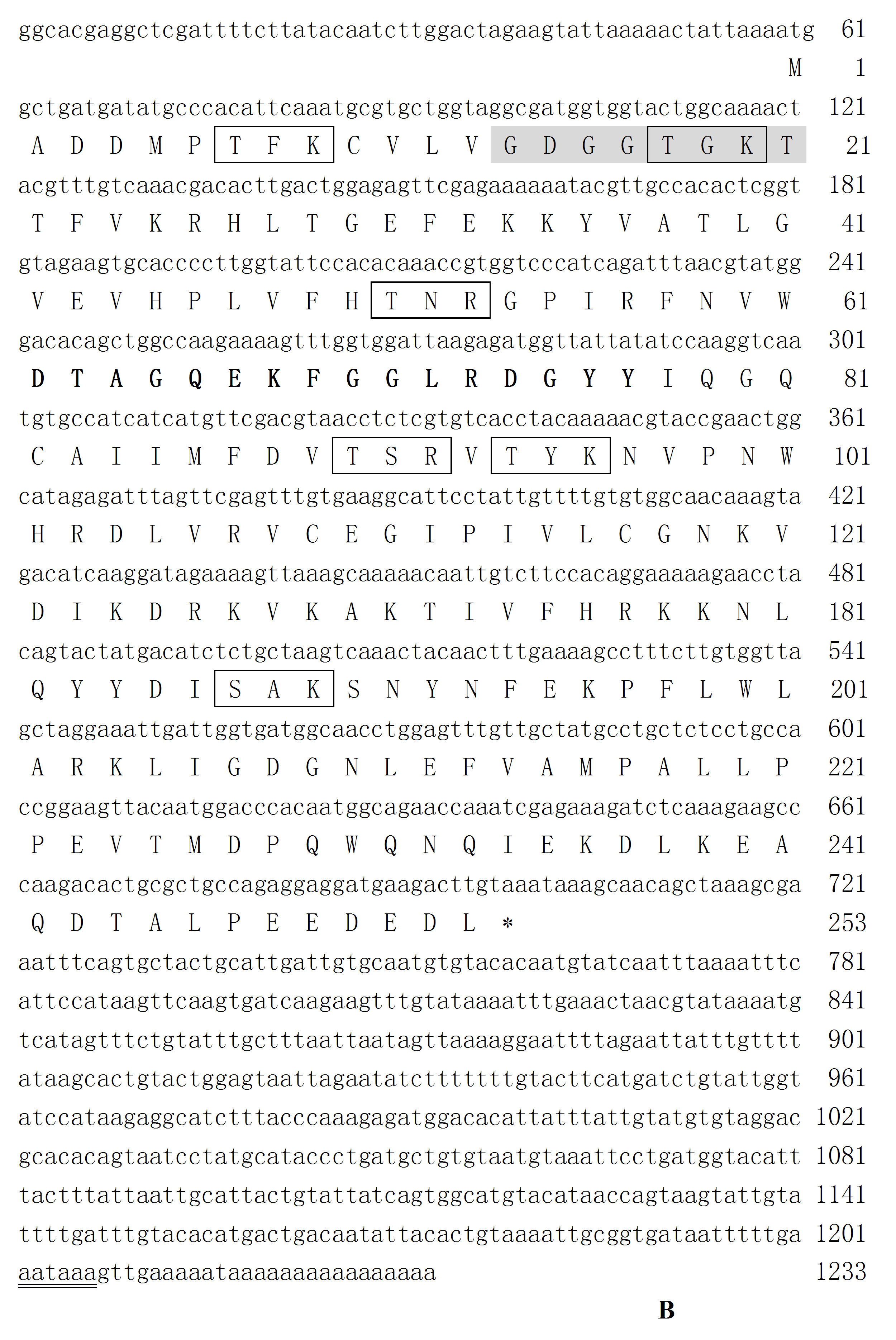

Supplement: Additional file 2 — Full-length cDNA sequences and predicted amino acid sequences of Ha-Ran. Full-length cDNA sequences and predicted amino acid sequences of Ha-Ran. Overstriking amino acid residues are GTP-binding nuclear protein Ran signatures; Boxed amino acid residues are protein kinase C phosphorylation sites. The shadowed sequence is an ATP/GTP-binding site motif A. Poly A tail adding signal is in broad brush. [file 1471-2121-11-1-S2.TIFF]

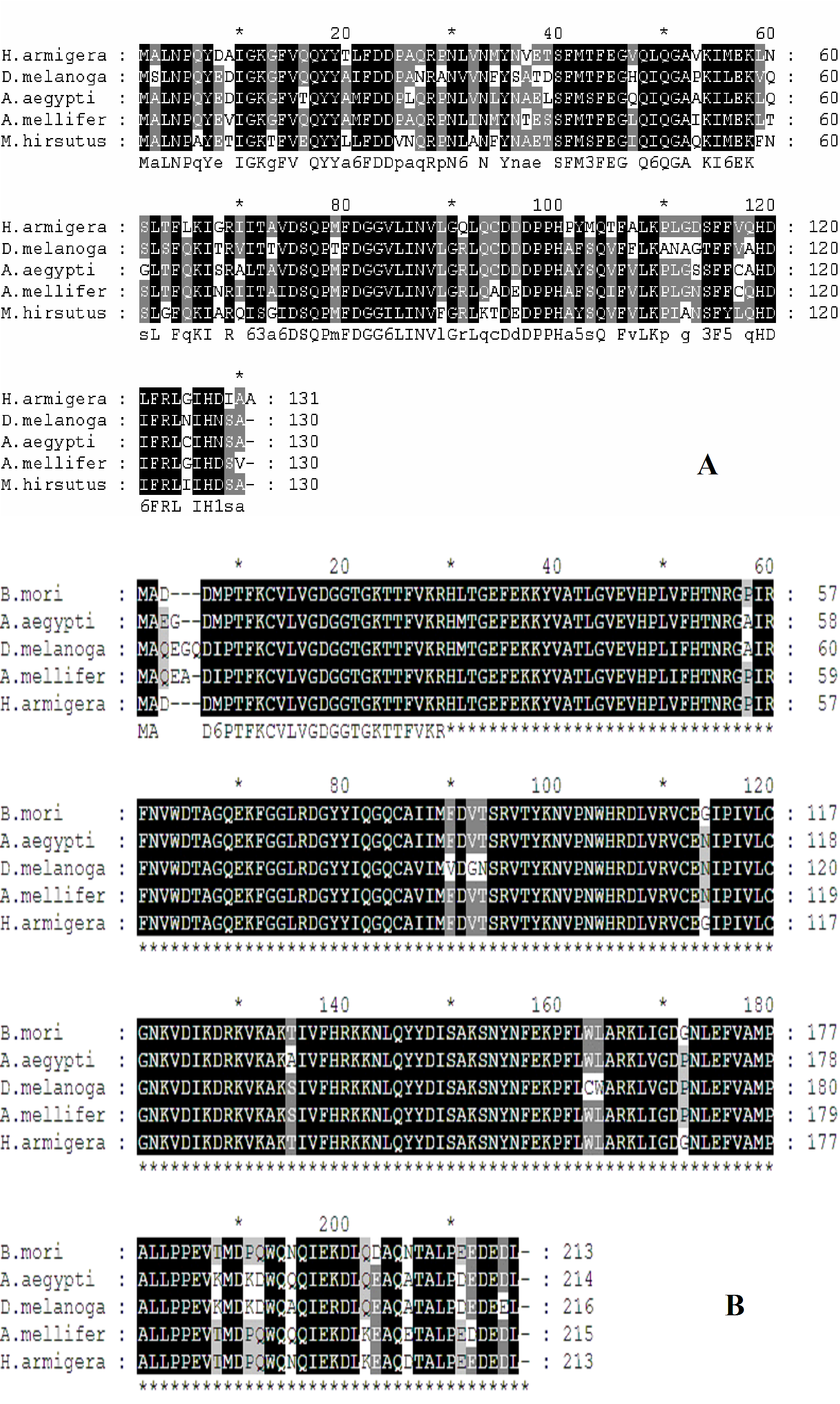

Supplement: Additional file 3 — Multiple alignments of Ha-Ntf2 and Ha-Ran with other insects. Multiple alignments of Ha-Ntf2 and Ha-Ran with other insects. D. melanogaster [Ntf2, AAS98195.1; Ran: NP_651969.1]; A. aegypti [Ntf2, AAS79346.1; Ran, EAT38849.1]; A. mellifera [Ntf2, XP_392921.1; Ran, XP_393761.1]; M. hirsutus [ABM55654.1]; B. mori [NP_001040274.1]; H. armigera [Ntf2, DQ875254; Ran, EU860296]. The shadow regions in black are conserved sequences. [file 1471-2121-11-1-S3.TIFF]

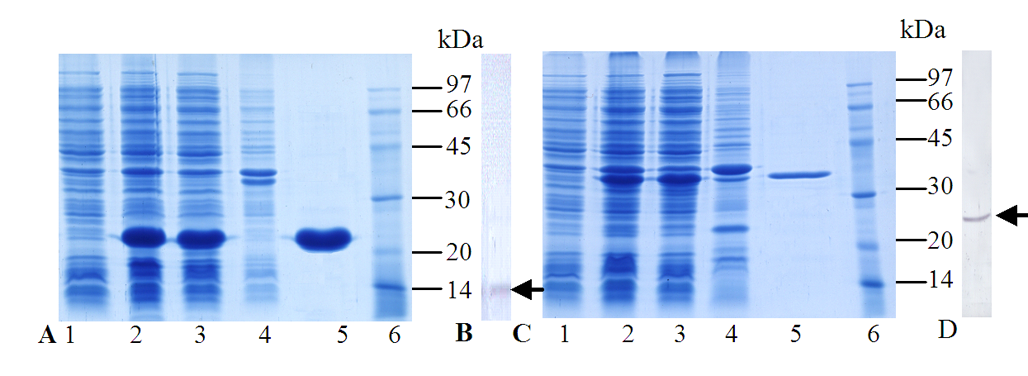

Supplement: Additional file 4 — Recombinant expression of Ha-Ntf2 and Ha-Ran in E. coli and specificity of antibodies. Recombinant expression of Ha-Ntf2 and Ha-Ran in E. coli and specificity of antibodies. A and C, analysis of recombinant expression of Ha-Ntf2 and Ha-Ran by 12.5% SDS-PAGE; B and D, examining the specificity of the antibody against Ha-Ntf2 and Ha-Ran by immunoblotting, arrow indicate the target protein from 5-36 h fat bodies. Lane 1, E. coli proteins with pET30a-Ha-Ntf2 or pET30a-Ha-Ran without induction; lane 2, E. coli proteins with pET30a-Ha-Ntf2 or pET30a-Ha-Ran induced by IPTG; lane 3, lysate supernate; lane 4, lysate precipitate; lane 5, purified recombinant protein by nickel affinity chromatography; lane 6, standard protein marker. [file 1471-2121-11-1-S4.TIFF]
